# Network pharmacology-based analysis and experimental in vitro validation on the mechanism of *Paeonia lactiflora* Pall. in the treatment for type I allergy

**DOI:** 10.1186/s12906-022-03677-z

**Published:** 2022-07-25

**Authors:** Yang Zhao, Hui Li, Xiangsheng Li, Yizhao Sun, Yuxin Shao, Yanfen Zhang, Zhongcheng Liu

**Affiliations:** 1 College of Pharmaceutical Sciences, Key Laboratory of Pharmaceutical Quality Control of Hebei Province, Institute of Life Science and Green Development, Hebei University, Baoding, 071002 People’s Republic of China; 2 Department of Urology, Peking University International Hospital, Beijing, 102206 People’s Republic of China; 3 Technology Transfer Center, Hebei University, Baoding, 071002 People’s Republic of China

**Keywords:** Traditional Chinese medicine, Allergic reaction, IgE/FcεR I

## Abstract

**Background:**

The incidence of allergic reaction is increasing year by year, but the specific mechanism is still unclear. *Paeonia lactiflora* Pall.(PLP) is a traditional Chinese medicine with various pharmacological effects such as anti-tumor, anti-inflammatory, and immune regulation. Previous studies have shown that PLP has potential anti-allergic activity. However, there is still no comprehensive analysis of the targeted effects and exact molecular mechanisms of the anti-allergic components of PLP. This study aimed to reveal the mechanism of PLP. in the treatment of type I allergy by combining network pharmacological methods and experimental verification.

**Methods:**

First, we used the traditional Chinese medicine systems pharmacology (TCMSP) database and analysis platform to screen the main components and targets of PLP, and then used databases such as GeneCards to retrieve target information related to ‘allergy’. Protein–protein interaction (PPI) analysis obtained the core target genes in the intersection target, and then imported the intersection target into the David database for gene ontology (GO) and Kyoto encyclopedia of genes and genomes (KEGG) analysis. Furthermore, the therapeutic effect of paeoniflorin, the main component of PLP, on IgE-induced type I allergy was evaluated in vitro.

**Results:**

GO analysis obtained the main biological processes, cell components and molecular functions involved in the target genes. KEGG analysis screened out MAPK1, MAPK10, MAPK14 and TNF that have a strong correlation with PLP anti-type I allergy, and showed that PLP may pass through signal pathways such as IgE/FcεR I, PI3K/Akt and MAPK to regulate type I allergy. RT-qPCR and Western Blot results confirmed that paeoniflorin can inhibit the expression of key genes and down-regulate the phosphorylation level of proteins in these signal pathways. It further proved the reliability of the results of network pharmacology research.

**Conclusion:**

The results of this study will provide a basis for revealing the multi-dimensional regulatory mechanism of PLP for the treatment of type I allergy and the development of new drugs.

**Supplementary Information:**

The online version contains supplementary material available at 10.1186/s12906-022-03677-z.

## Background

The incidence and mortality of allergic diseases is increasing, and has become a common disease, which greatly affects people's life and physical health. But so far, people have not revealed its exact pathogenesis, and there is no ideal treatment method. At present, glucocorticoid and antihistamine are commonly used clinically to treat allergy, but the curative effect is short and there are many adverse reactions after long-term use [1]. Therefore, it is necessary to continue to explore effective and safe new methods to treat allergic diseases. Last several years, the advantages of traditional Chinese medicine (TCM) with multiple targets and curative effects, and less adverse reactions have attracted the attention of many researchers. TCM is becoming a hot spot in the research and development of drugs to treat allergic diseases [2].

TCM has been used for the treatment of allergic diseases with long history. But due to the complex chemical components and pharmacological effects of TCM, its specific effective substance basis and mechanism are still unclear, which brings huge challenges to the study of the mechanism of TCM to treat allergy. For the past few years, with the in-depth research of TCM and the development of related technologies, the use of TCM in treatment of allergic diseases has gained great recognition and breakthroughs. It has been found that many TCMs and their components have therapeutic effects on allergy, such as Polydatin, Glycyrrhizic acid and Quercetin [3–5]. Treasury of TCM has huge potential for new drug research, and shows excellent application prospects to treat allergy. However, there is still a great deal of potential TCMs with anti-allergic activity waiting to be explored, such as PLP.

The medicinal part of PLP is its dried root, and it has many pharmacological effects such as protecting liver, nerve and heart, anti-tumor, anti-inflammatory and immune regulation. The main active ingredient of PLP is Paeoniflorin (Pae) [6, 7]. Studies have confirmed that PLP and Pae have potential anti-allergic activity [8, 9]. In view of the complexity of the cell signal network involved in allergy, these conclusions should be part of the mechanism for its effectiveness. So the molecular mechanism and specific biological process of PLP anti-allergy still need to be further elucidated. The purpose of this study was to explore the regulation mechanism of multiple genes and multiple pathways in the treatment of type I allergy with PLP.

Network pharmacology is a research method based on multi-directional pharmacology and systems biology, which can analyze the relationship between drugs and diseases at the overall level. Network pharmacology is based on the drug-target-disease network, so as to systematically explore the specific mechanisms of drug to treat diseases. Its greatest advantage is the integration of holistic, dynamic and analysis, which is consistent with the holistic and dialectical treatment principles of TCM [10].

Consequently, our research was based on the network pharmacology to systematically analyze the active ingredients of PLP, allergy-related targets and their pathways to identify potential drug targets and mechanisms. Type I allergy is the most common type of allergy in clinical practice. We used cell models and in vitro experiments to explore the effects and related mechanisms of Pae, the main active ingredient of PLP, in treating type I allergy. Most reports on the relationship between Pae and allergy only focused on showing the inhibitory effects of this compound and lacked in-depth exploration of the underlying mechanism [11, 12].Therefore, in this study, the combined approaches offer deep understanding of the pharmacological mechanisms of PLP, and may provide a novel and efficient way to discover the pharmacological basis and medicinal value of PLP.

## Materials and methods

### Materials

RBL-2H3 cells were obtained from the ATCC. PrimeScript™ RT reagent Kit, TB Green Kit were purchased from Takara (Beijing, China). The finished product of Paeoniflorin (HPLC ≥ 98%, and is usually extracted from the root of PLP) were purchased from Solarbio (Beijing, China).

### Network pharmacology analysis

#### Screening of the main active ingredients of PLP and acquisition of its targets

Traditional Chinese Medicine Systems Pharmacology Database and Analysis Platform (TCMSP) is a database established based on the framework of TCM system pharmacology, providing 12 important pharmacokinetic properties, such as oral bioavailability (OB) and drug-likeness (DL), which is mainly used to screen and evaluation of pharmaceutical compounds. OB is an important indicator for evaluating whether a drug can be developed, and OB ≥ 30% is considered to have better oral bioavailability. DL can evaluate the possibility of a compound becoming a drug, and DL ≥ 0.18 is considered to have high drug-likeness and may become a new drug [13]. Our method and operation were carried out with reference to relevant literature [14, 15], and the specific steps were as follows: The PLP was imported into the TCMSP database (https://tcmspw.com/tcmsp.php), and all known chemical components contained in the PLP have been retrieved and screened for potential activities, that is, OB ≥ 30%, DL ≥ 0.18. According to the active ingredients obtained after screening, the TCMSP database is used again to retrieve its target.

#### Acquisition of targets for allergy

The GeneCards (https://www.genecards.org/) database is not only a database that can provide concise genome, proteome, transcription, inheritance and function of all known and predicted human genes, but also an analytical database that combines retrieval, integration, search and display of the information of the human genome [16]. The OMIM database (http://omim.org/) catalogs the genetic components of all known diseases and links them with related genes in the human genome when possible. It provides a reference for further research and genomic analysis tools of cataloging genes [17]. In these two databases, searched with ‘allergy’ as a keyword to find the target of allergy.

#### Establishment and analysis of protein–protein interaction (PPI) network

Used the Draw Venn database (http://bioinformatics.psb.ugent.be/webtools/Venn/) to take the intersection of the targets obtained in 2.2.1 and 2.2.2, and imported it into the String database (https://string-db.org/). Then used ‘Multiple proteins’ function to establish the PPI network, selected the species as ‘Homo sapiens’, and clicked ‘SEARCH’ and ‘CONTINUE’ options to get the PPI network.

#### Analysis of biological processes and pathway enrichment

The David database (https://david.ncifcrf.gov/) can be used for enrichment analysis of a great quantity of sample genes and proteins, also can simultaneously provide systematic and comprehensive biological information. Through the integration and analysis of information, we can intuitively show the pathway enrichment of target genes, which has become one of the indispensable tools of bioinformatics research. Imported the target obtained in 2.2.3 into the David database for Gene Ontology (GO) analysis and Kyoto Encyclopedia of Genes and Genomes (KEGG) analysis. GO analysis is a description of genes in different dimensions and levels, which includes three aspects: biological process (BP), cell component (CC) and molecular function (MF). KEGG is a database that links gene catalogs obtained from fully sequenced genomes with system functions of higher-level cell, species, and ecosystem. KEGG analysis discovers the pathways of drug targets by enriching target genes, thereby obtaining the mechanism of drug treatment of diseases [18]. Selected the species as ‘Homo sapiens’, and conducted target analysis through MF, BP, and CC in GO. Simultaneously selected KEGG in Pathway for pathway analysis, and screened the results with the -LogP ≥ 2 for analysis.

#### Network establishment

Cytoscape is a mapping software that can be used to establish, analyze, and visualize complex networks. It is often used to analyze the results of network pharmacology. Used Excel to establish data sets of PLP-signal pathway and signal pathway-target, and imported them into Cytoscape to establish the network of PLP-target-signal pathway.

### In vitro experiments

#### Western Blot analysis

Our experimental method was performed with reference to relevant literature [19, 20], and the specific steps were as follows: After culturing RBL-2H3 cells (5 × 10^5^ cells/mL) for 24 h, each group was sensitized with 1 mL of DNP-IgE (0.2 μg/mL). After 12 h, drug groups were replaced with 2 mL of the corresponding drug respectively (Pae 0.5, 2.5, 5 μg/mL, Keto 25 μg/mL). After 1 h, in addition to the normal group, 400 μL of DNP-BSA (0.4 μg/mL) was added for stimulation. After 30 min, extracted total protein and measured its concentration.

The experiment used 8% separating gel, 4% stacking gel, and loaded 30 μg protein sample. After electrophoresis, the cut gel was transferred to the PVDF membrane. The PVDF membrane was blocked with shaking at room temperature for 1 h. After incubation with primary antibodies of Lyn, p-Lyn, Syk, p-Syk and β-actin at 4℃ overnight, the secondary antibodies were incubated at room temperature for 1 h. Visualization was performed by using the ChemiScope Mini 3300 and density analysis was performed with Image J software.

#### qPCR

The steps were the same as 2.3.1. Then extracted total RNA, removed gDNA from RNA and performed reverse transcription by using PrimeScript™ RT reagent Kit. Used TB Green kit for qPCR reaction. The key genes tested include: Lyn, Syk, Fyn, PLCγ, PI3K, Akt, p38, ERK, JNK, p65 and GAPDH.

### Statistical analysis

Results were expressed as the mean ± SD. ANOVA in SPSS 17.0 software was used to assess significant differences between groups (*p* < 0.05).

## Results

### Main active ingredients of PLP and its targets

As shown in Table 1, there are 29 main active ingredients of PLP, including Pae, and 157 targets obtained from the TCMSP database.

**Table 1:** The main active ingredients of PLP

	Mol ID	Molecule Name	OB (%)	DL
1	MOL001002	ellagic acid	43.06	0.43
2	MOL001918	paeoniflorgenone	87.59	0.37
3	MOL001921	Lactiflorin	49.12	0.8
4	MOL001924	paeoniflorin	53.87	0.79
5	MOL001925	paeoniflorin_qt	68.18	0.4
6	MOL002714	baicalein	33.52	0.21
7	MOL002776	Baicalin	40.12	0.75
8	MOL000358	beta-sitosterol	36.91	0.75
9	MOL000359	sitosterol	36.91	0.75
10	MOL004355	Spinasterol	42.98	0.76
11	MOL000449	Stigmasterol	43.83	0.76
12	MOL000492	( +)-catechin	54.83	0.24
13	MOL006990	(1S,2S,4R)-trans-2-hydroxy-1,8-cineole-B-D-glucopyranoside	30.25	0.27
14	MOL006992	(2R,3R)-4-methoxyl-distylin	59.98	0.3
15	MOL006994	1-o-beta-d-glucopyranosyl-8-o-benzoylpaeonisuffrone_qt	36.01	0.3
16	MOL006996	1-o-beta-d-glucopyranosylpaeonisuffrone_qt	65.08	0.35
17	MOL006999	stigmast-7-en-3-ol	37.42	0.75
18	MOL007003	benzoyl paeoniflorin	31.14	0.54
19	MOL007004	Albiflorin	30.25	0.77
20	MOL007005	Albiflorin_qt	48.7	0.33
21	MOL007008	4-ethyl-paeoniflorin_qt	56.87	0.44
22	MOL007012	4-o-methyl-paeoniflorin_qt	56.7	0.43
23	MOL007014	8-debenzoylpaeonidanin	31.74	0.45
24	MOL007016	Paeoniflorigenone	65.33	0.37
25	MOL007018	9-ethyl-neo-paeoniaflorin A_qt	64.42	0.3
26	MOL007022	evofolinB	64.74	0.22
27	MOL007025	isobenzoylpaeoniflorin	31.14	0.54
28	MOL002883	Ethyl oleate (NF)	32.4	0.19
29	MOL005043	campest-5-en-3beta-ol	37.58	0.71

### Target of allergy

Through GeneCards and OMIM database searched, 2424 targets related to ‘allergy’ were obtained (Too much data to show).

### Analysis of PPI network

**Fig. 1:**
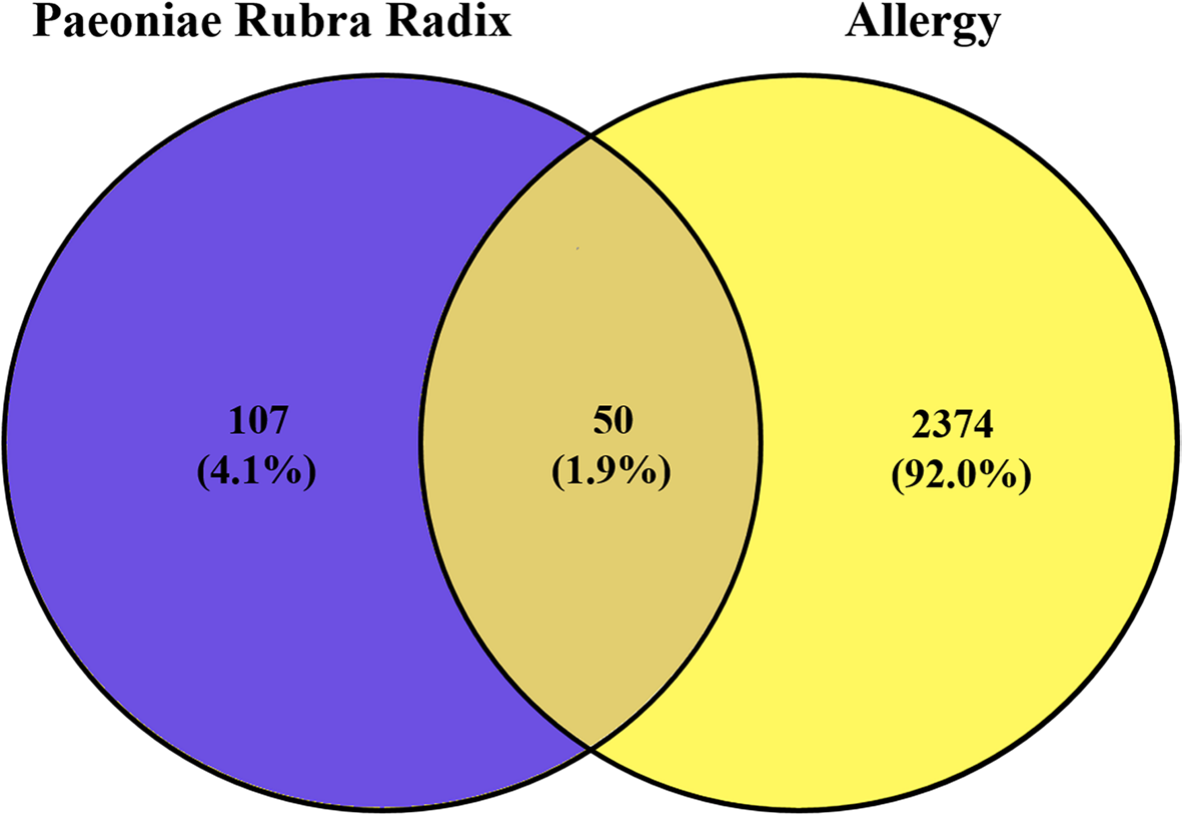
Intersection of the targets of PLP and allergy

**Fig. 2:**
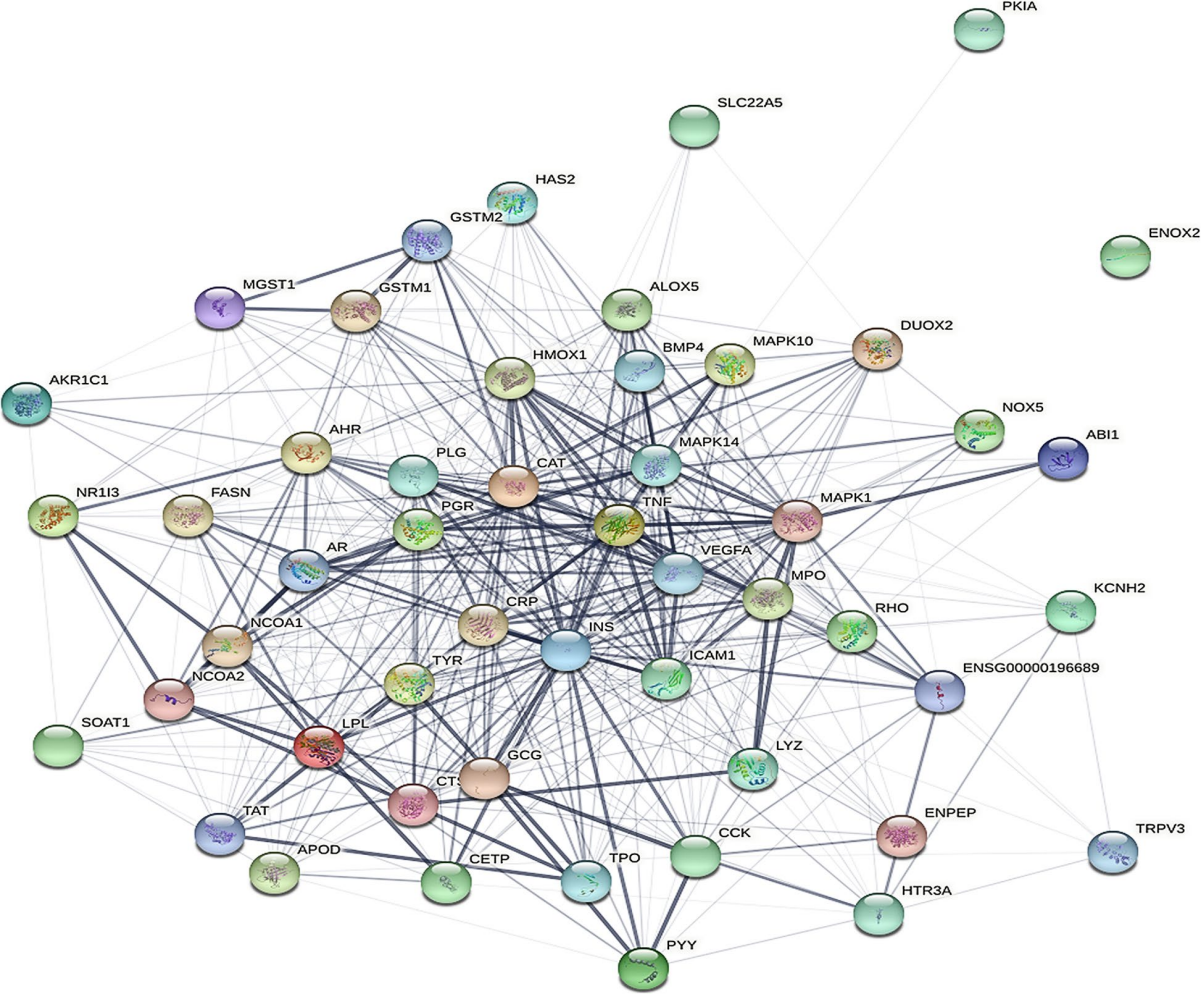
PPI network of PLP-allergy target

**Fig. 3:**
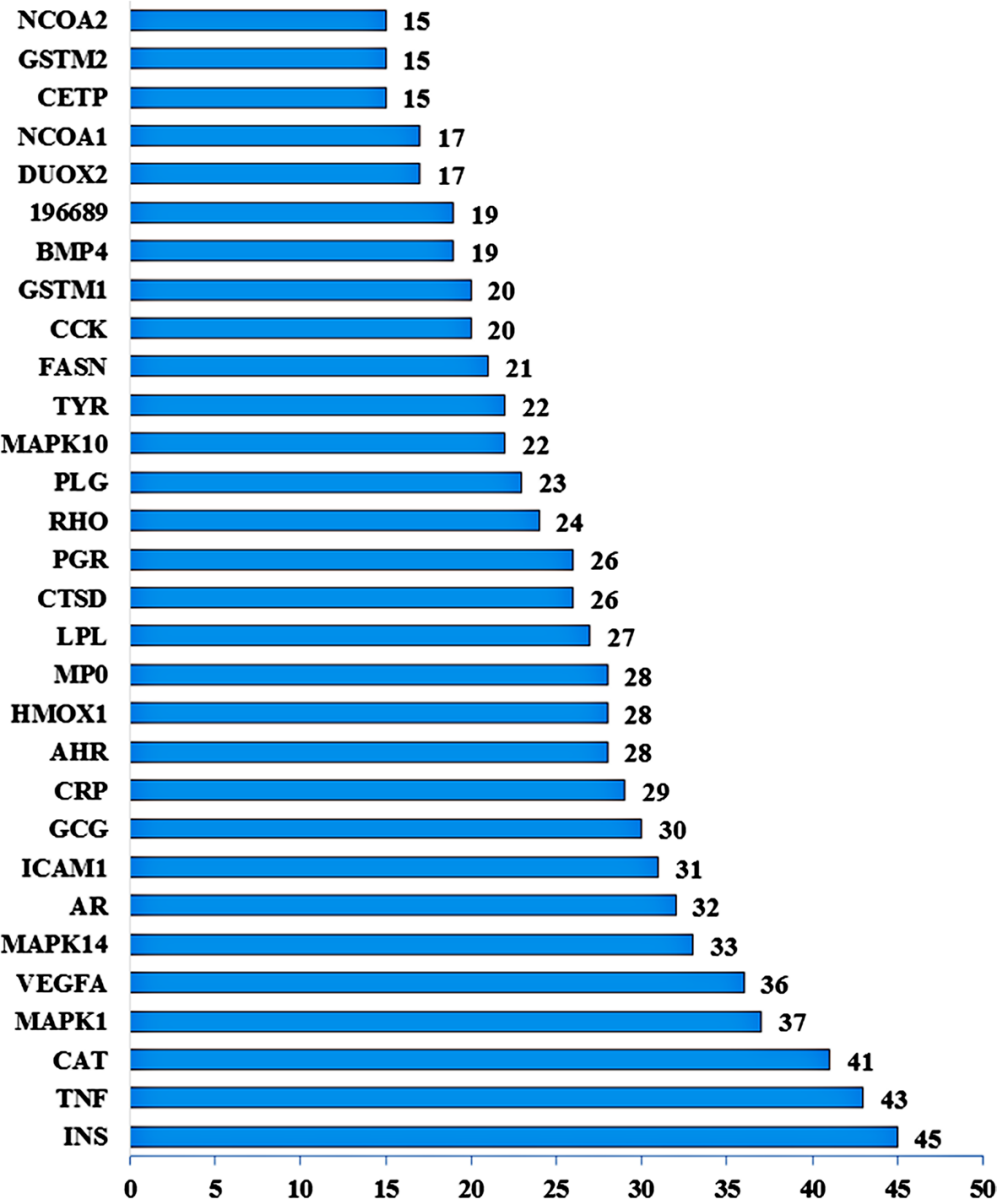
PPI network of top 30 target genes

Imported the two target sets obtained in 2.2.1 and 2.2.2 into the Draw Venn database to obtain the intersection (Fig. 1). It is found that there are 50 potential targets of PLP in allergy (as shown in Table 2), which were imported into the String database to establish PPI (as shown in Fig. 2), among which the top 5 interaction relationships according to the number are: INS, TNF, CAT, MAPK1 and VEGFA (Fig. 3).

**Table 2:** The potential targets of PLP in allergy

	Target name	Gene Symbol
1	androgen receptor	AR
2	progesterone receptor	PGR
3	vascular endothelial growth factor a	VEGFA
4	glutathione s-transferase mu 1	GSTM1
5	transient receptor potential cation channel subfamily v member 1	TRPV1
6	arachidonate 5-lipoxygenase	ALOX5
7	catalase	CAT
8	plasminogen	PLG
9	thyroid peroxidase	TPO
10	tumor necrosis factor	TNF
11	myeloperoxidase	MPO
12	aryl hydrocarbon receptor	AHR
13	potassium voltage-gated channel subfamily h member 2	KCNH2
14	5-hydroxytryptamine receptor 3a	HTR3A
15	mitogen-activated protein kinase 14	MAPK14
16	cathepsin d	CTSD
17	solute carrier family 22 member 5	SLC22A5
18	mitogen-activated protein kinase 1	MAPK1
19	intercellular adhesion molecule 1	ICAM1
20	tyrosinase	TYR
21	c-reactive protein	CRP
22	insulin	INS
23	glucagon	GCG
24	cholecystokinin	CCK
25	cholesteryl ester transfer protein	CETP
26	peptide yy	PYY
27	nuclear receptor subfamily 1 group i member 3	NR1I3
28	hemeoxygenase 1	HMOX1
29	glutathione s-transferase mu 2	GSTM2
30	lysozyme	LYZ
31	nuclear receptor coactivator 2	NCOA2
32	fatty acid synthase	FASN
33	aldo-ketoreductase family 1 member c1	AKR1C1
34	tyrosine aminotransferase	TAT
35	nuclear receptor coactivator 1	NCOA1
36	nadph oxidase 5	NOX5
37	apolipoprotein d	APOD
38	hyaluronan synthase 2	HAS2
39	microsomal glutathione s-transferase 1	MGST1
40	rhodopsin	RHO
41	transient receptor potential cation channel subfamily v member 3	TRPV3
42	dual oxidase 2	DUOX2
43	mitogen-activated protein kinase 10	MAPK10
44	ablinteractor 1	ABI1
45	lipoprotein lipase	LPL
46	sterol o-acyltransferase 1	SOAT1
47	bone morphogenetic protein 4	BMP4
48	camp-dependent protein kinase inhibitor alpha	PKIA
49	ecto-nox disulfide-thiol exchanger 2	ENOX2
50	glutamylaminopeptidase	ENPEP

### Analysis of biological process and pathway enrichment

**Fig. 4:**
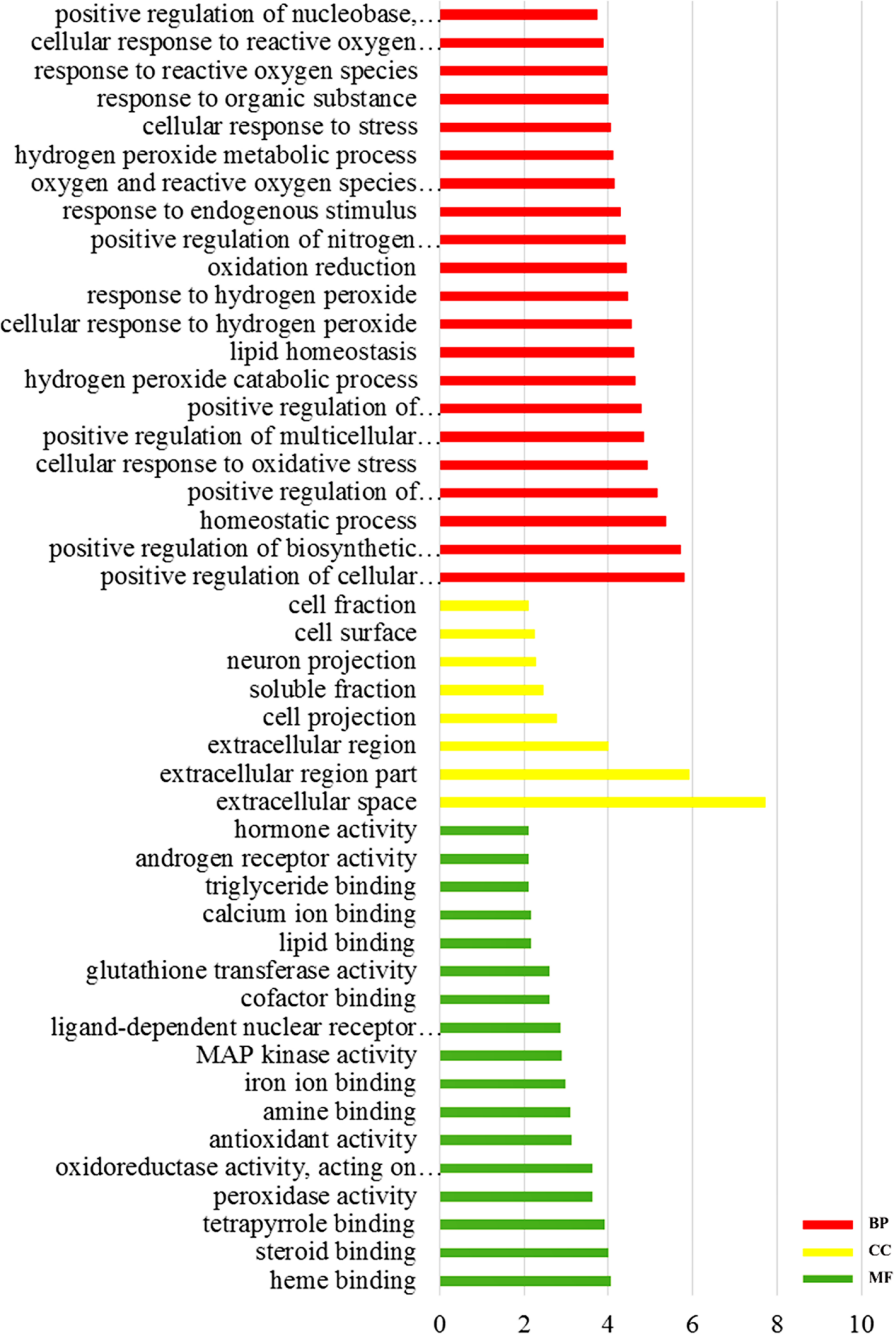
Results of GO analysis

Imported the obtained 50 intersection targets into the David database for GO and KEGG analysis. As shown in Table 3, GO-BP analysis obtained 235 results of PLP anti-allergic effects, 109 of them are -LogP ≥ 2, and the biological processes with the number of genes ≥ 18 are mainly: positive regulation of cell biosynthesis process, positive regulation of polymer biosynthesis and metabolic process, redox, regulation of cell death and apoptosis, transcription regulation, regulation of RNA metabolic process, intracellular signal cascade and so on. GO-CC analysis obtained 27 results, 8 of them are -LogP ≥ 2, and these cell locations with the number of genes ≥ 10 mainly include the extracellular region and the plasma membrane. GO-MF analysis obtained 41 results, and 17 of them are -LogP ≥ 2. The molecular processes involved are antioxidant activity, MAPK activity, binding of Ca^2+^ and triglycerides and so on. The process with the number of genes ≥ 10 is binding of Ca^2+^. The visual processing was showed in Fig. 4.

**Table 3:** GO analysis of anti-allergic reactions of PLP

	Name	-LogP
BP	positive regulation of cellular biosynthetic process	5.799403
BP	positive regulation of biosynthetic process	5.733428
BP	homeostatic process	5.383481
BP	positive regulation of macromolecule biosynthetic process	5.155981
BP	positive regulation of macromolecule metabolic process	4.799018
BP	oxidation reduction	4.436033
BP	positive regulation of nitrogen compound metabolic process	4.407209
BP	cellular response to stress	4.071929
BP	response to organic substance	3.994682
BP	positive regulation of nucleobase, nucleoside, nucleotide and nucleic acid metabolic process	3.749874
BP	regulation of cell death	3.558830
BP	regulation of transcription from RNA polymerase II promoter	3.25929
BP	regulation of apoptosis	2.946298
BP	regulation of programmed cell death	2.915996
BP	regulation of transcription, DNA-dependent	2.394503
BP	regulation of RNA metabolic process	2.310271
BP	intracellular signaling cascade	2.143719
BP	regulation of transcription	2.129554
CC	extracellular space	7.734715
CC	extracellular region part	5.92365
CC	extracellular region	3.996677
CC	cell projection	2.76938
CC	soluble fraction	2.461935
CC	neuron projection	2.298664
CC	cell surface	2.26695
CC	cell fraction	2.123144
MF	heme binding	4.051026
MF	steroid binding	4.016238
MF	tetrapyrrole binding	3.919531
MF	peroxidase activity	3.634473
MF	oxidoreductase activity, acting on peroxide as acceptor	3.634473
MF	antioxidant activity	3.137301
MF	amine binding	3.113086
MF	iron ion binding	2.997328
MF	MAP kinase activity	2.908816
MF	ligand-dependent nuclear receptor activity	2.870518
MF	cofactor binding	2.621835
MF	glutathione transferase activity	2.595378
MF	lipid binding	2.177942
MF	calcium ion binding	2.170265
MF	triglyceride binding	2.122953
MF	androgen receptor activity	2.122953
MF	hormone activity	2.107126

**Fig. 5:**
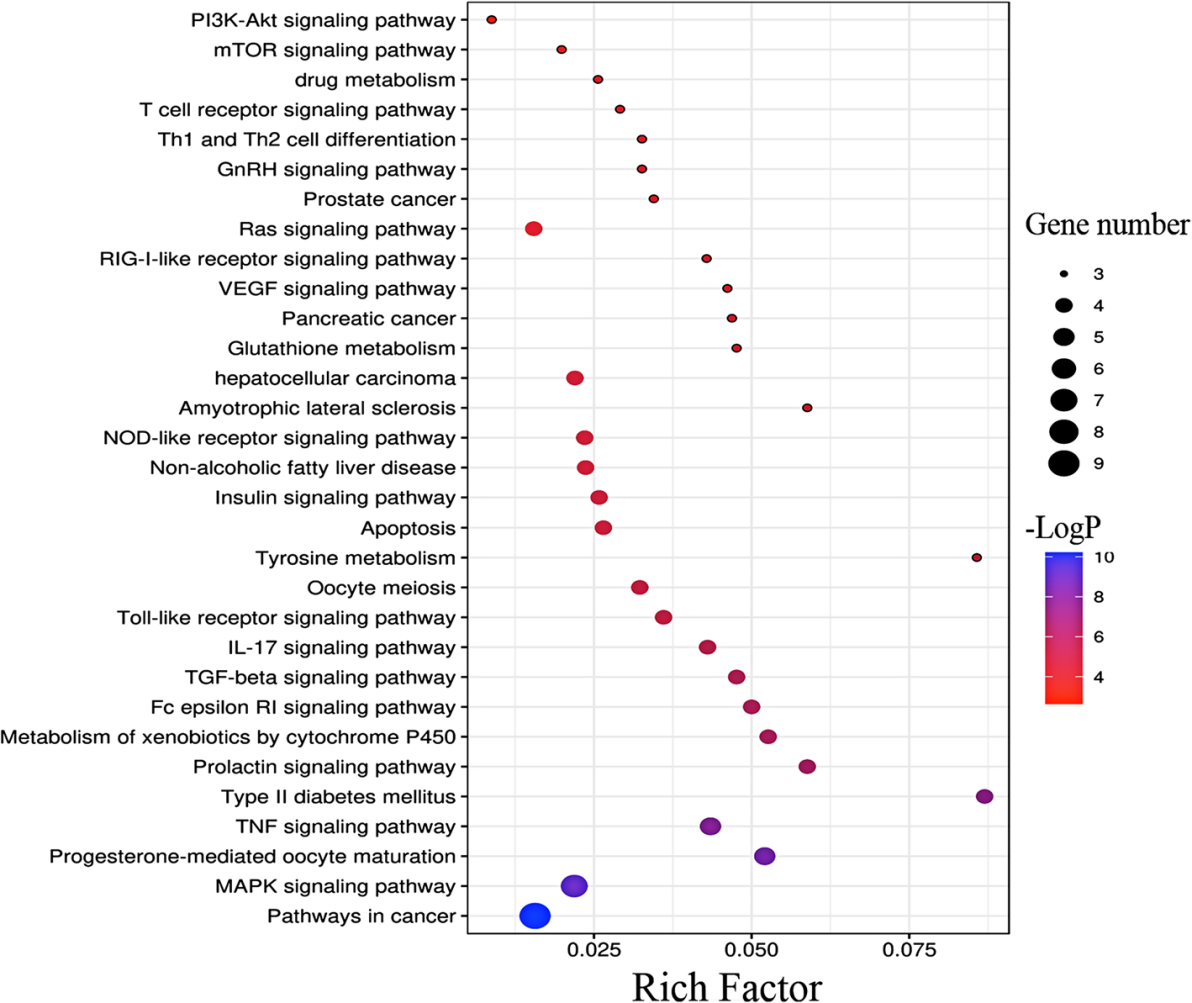
Enrichment analysis of pathways

**Fig. 6:**
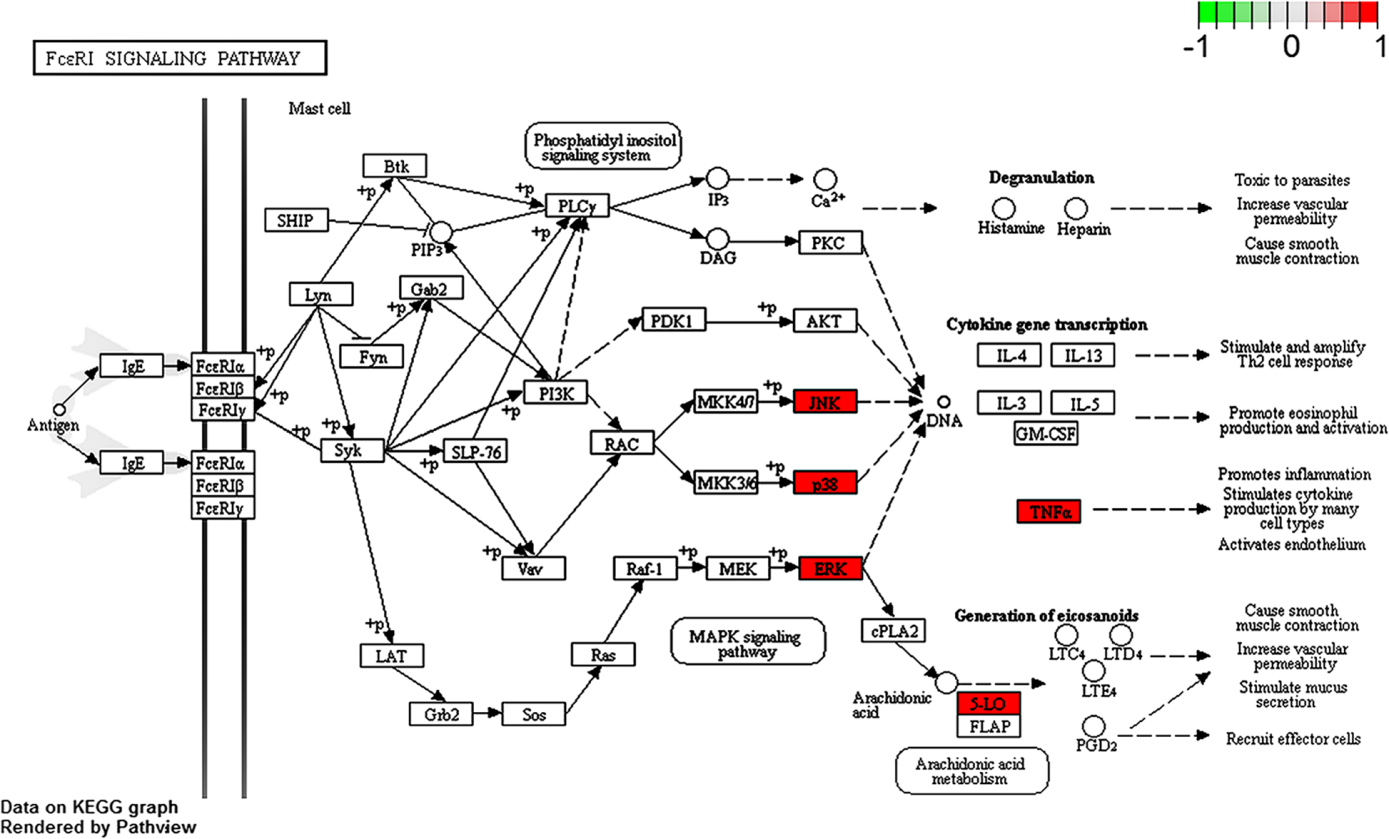
Important target genes are mainly distributed in the FcεR I signal pathway

Through KEGG analysis, 31 related pathways were obtained (Table 4). The top 13 signal pathways according to the number of genes mainly include: tumor-related signal pathway, MAPK signal pathway, TNF signal pathway, liver cancer signal pathway, type II diabetes-related signal pathway, lactation signal pathway, FcεR I signal pathway and IL-17 signal pathway. In addition, the anti-allergic effect of PLP may also be related to Th cell differentiation and PI3K/Akt signal pathway. Visualized the above-mentioned signal pathways with the Metascape database (http://metascape.org/gp/index.html), and obtained the bubble chart of related pathways of PLP anti-allergic effect (Fig. 5), in which the values of Rich Factor and -LogP both are positively correlated with the degree of enrichment. Moreover, the important targets of MAPK 1, MAPK 10, MAPK 14 and TNF are mainly distributed in the FcεR I signal pathway that is related to allergic reaction (Fig. 6, and the copyright of this KEGG pathway picture belongs to Kanehisa Laboratory).

**Table 4:** KEGG analysis of anti-allergic reactions of PLP

	Pathway name	-LogP	Gene number
1	Pathways in cancer	10.3275	9
2	MAPK signaling pathway	8.970662	7
3	Progesterone-mediated oocyte maturation	8.320762	5
4	TNF signaling pathway	7.92438	5
5	Metabolism of xenobiotics by cytochrome P450	6.724768	4
6	hepatocellular carcinoma	5.207091	4
7	Type II diabetes mellitus	7.614715	4
8	Prolactin signaling pathway	6.920665	4
9	Fc epsilon RI signaling pathway	6.634641	4
10	IL-17 signaling pathway	6.370812	4
11	Toll-like receptor signaling pathway	6.062156	4
12	Apoptosis	5.528667	4
13	Insulin signaling pathway	5.483541	4
14	Non-alcoholic fatty liver disease	5.33452	4
15	NOD-like receptor signaling pathway	5.324366	4
16	Ras signaling pathway	4.60425	4
17	TGF-beta signaling pathway	6.549032	4
18	Oocyte meiosis	5.869675	4
19	Glutathione metabolism	4.988936	3
20	drug metabolism	4.182712	3
21	Pancreatic cancer	4.968272	3
22	VEGF signaling pathway	4.947937	3
23	RIG-I-like receptor signaling pathway	4.850846	3
24	Th1 and Th2 cell differentiation	4.494327	3
25	GnRH signaling pathway	4.494327	3
26	T cell receptor signaling pathway	4.347688	3
27	mTOR signaling pathway	3.854131	3
28	PI3K-Akt signaling pathway	2.819689	3
29	Prostate cancer	4.567031	3
30	Tyrosine metabolism	5.766723	3
31	Amyotrophic lateral sclerosis	5.267027	3

### Network of PLP-target-signal pathway

**Fig. 7:**
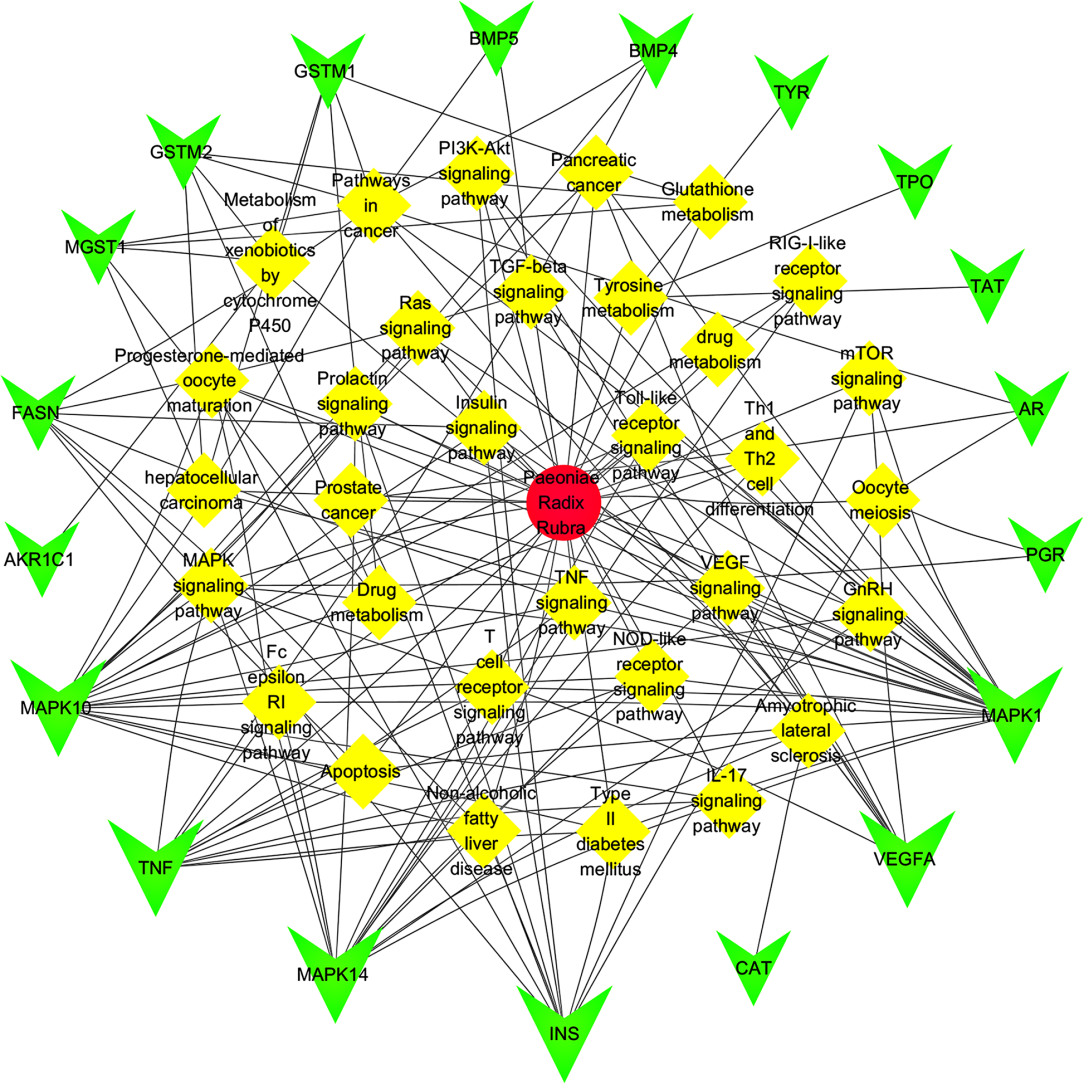
Network of PLP-target-pathway

Cytoscape was used for establish the network of PLP-target-signal pathway (Fig. 7). Red represents PLP, yellow represents signal pathway, and green represents intersection target. There are 52 nodes and 153 edges in this figure. In topological metrics analysis, node centrality is a widely used measurement with three main metrics: degree, closeness, and betweeness. These three topological metrics were selected as candidate targets. After comprehensively analyzing the values of the three metrics for each target in this network, it was found that the top four targets were MAPK 1, MAPK 10, MAPK 14 and TNF (Table 5). Therefore, they are considered as important candidate targets of PLP for the treatment of allergy.

**Table 5:** Topological metrics analysis of network

	Nude	Degree	Closeness	Betweeness
1	MAPK 1	24	0.53125	0.15264486
2	MAPK 10	18	0.47222222	0.06938654
3	MAPK 14	14	0.4047619	0.03426261
4	TNF	12	0.3984375	0.02919505
5	Pathways in cancer	10	0.43965517	0.09264678
6	MAPK signaling pathway	10	0.49038462	0.06359126
7	INS	10	0.38059701	0.01816986
8	VEGFA	8	0.3984375	0.0178217
9	TGF-beta signaling pathway	7	0.46363636	0.04071533
10	FASN	7	0.39230769	0.01303181
11	TNF signaling pathway	6	0.45535714	0.01066349
12	Metabolism of xenobiotics by cytochrome P450	5	0.45535714	0.06255236
13	hepatocellular carcinoma	5	0.45535714	0.03820949
14	Oocyte meiosis	5	0.44736842	0.02674978
15	GSTM 1	5	0.36428571	0.01750897
16	GSTM 2	5	0.36428571	0.01750897
17	MGST 1	5	0.36428571	0.01750897
18	Non-alcoholic fatty liver disease	5	0.44736842	0.00885468
19	Insulin signaling pathway	5	0.44736842	0.00812833
20	Apoptosis	5	0.44736842	0.00743339
21	Type II diabetes mellitus	5	0.44736842	0.00686433
22	Progesterone-mediated oocyte maturation	5	0.44736842	0.00659734
23	Prolactin signaling pathway	5	0.44736842	0.00659734
24	Fc epsilon R I signaling pathway	5	0.44736842	0.00553748
25	IL-17 signaling pathway	5	0.44736842	0.00553748
26	Toll-like receptor signaling pathway	5	0.44736842	0.00553748
27	NOD-like receptor signaling pathway	5	0.44736842	0.00553748
28	Tyrosine metabolism	4	0.43220339	0.11529412
29	Amyotrophic lateral sclerosis	4	0.43220339	0.04152907
30	Glutathione metabolism	4	0.44736842	0.02333667
31	Prostate cancer	4	0.43965517	0.01329679
32	mTOR signaling pathway	4	0.43965517	0.00569641
33	PI3K-Akt signaling pathway	4	0.43965517	0.00569641
34	VEGF signaling pathway	4	0.43965517	0.00551107
35	Pancreatic cancer	4	0.43965517	0.00455056
36	Ras signaling pathway	4	0.43965517	0.00455056
37	T cell receptor signaling pathway	4	0.43965517	0.00387705
38	Th1 and Th2 cell differentiation	4	0.43965517	0.00311393
39	GnRH signaling pathway	4	0.43965517	0.00311393
40	AR	3	0.35915493	0.00368863
41	BMP 4	3	0.36956522	0.00260806
42	RIG-I-like receptor signaling pathway	3	0.43220339	0.00193453
43	Drug metabolism	3	0.27419355	4.71E-04
44	BMP 5	2	0.34931507	7.81E-04
45	PGR	2	0.34	5.39E-04
46	drug metabolism	1	0.41129032	0
47	AKR1C1	1	0.31481481	0
48	TYR	1	0.30357143	0
49	TPO	1	0.30357143	0
50	TAT	1	0.30357143	0
51	CAT	1	0.30357143	0

### Pae can inhibit the phosphorylation of Lyn and Syk proteins when RBL-2H3 cells degranulation

**Fig. 8:**
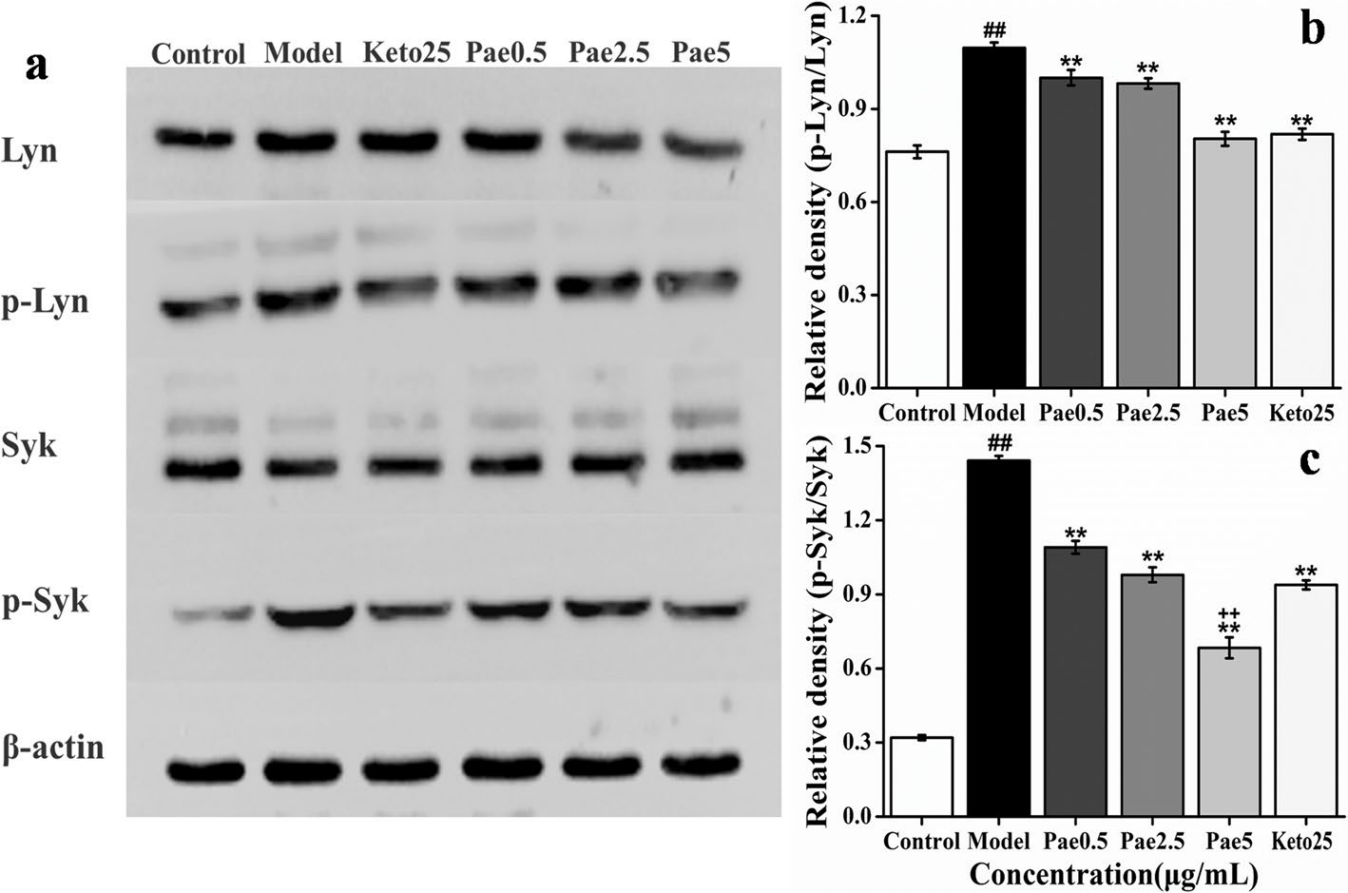
Effect of Pae on the phosphorylation of Lyn and Syk (*n* = 3). **a** Western Blot detected the phosphorylation of Lyn and Syk in RBL-2H3 cells. **b** Density analysis of Lyn. **c** Density analysis of Syk. ^##^*p* < 0.01 vs control; **p < 0.01 vs model; ^++^*p* < 0.01 vs Keto

Pae can inhibit the phosphorylation levels of Lyn and Syk proteins during the degranulation of RBL-2H3 cells in a dose-dependent manner (Fig. 8 and Additional file 1, 2, 3, 4, 5: Fig.S1-5). The inhibitory effect of 5 μg/mL Pae on phosphorylation of Syk protein was significantly stronger than positive control group (Keto group).

### Effect of Pae on the expression of genes when RBL-2H3 cells degranulation

**Fig. 9:**
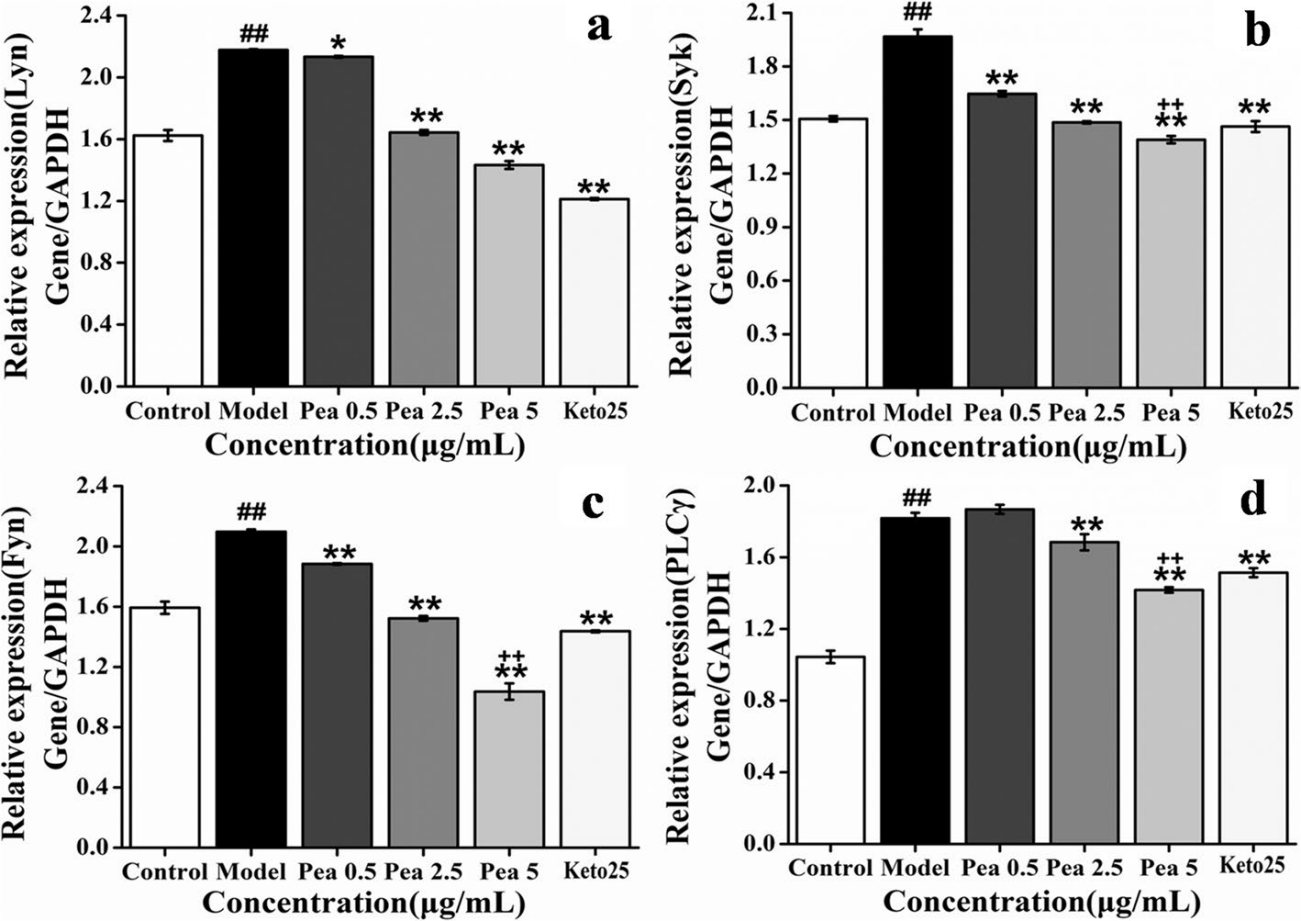
Effect of Pae on the expression of Lyn, Syk, Fyn and PLCγ in the IgE signal pathway (*n* = 3). **a** Lyn; **b** Syk; **c** Fyn; **d** PLCγ. ^##^*p* < 0.01 vs control; *p < 0.05, **p < 0.01 vs model; ^++^*p* < 0.01 vs Keto

Pae can inhibit the expression of Lyn, Syk, Fyn and PLCγ genes when the degranulation of RBL-2H3 cells in a dose-dependent manner (Fig. 9). The inhibitory effect of 5 μg/mL Pae on Syk, Fyn and PLCγ was stronger than Keto group.

**Fig. 10:**
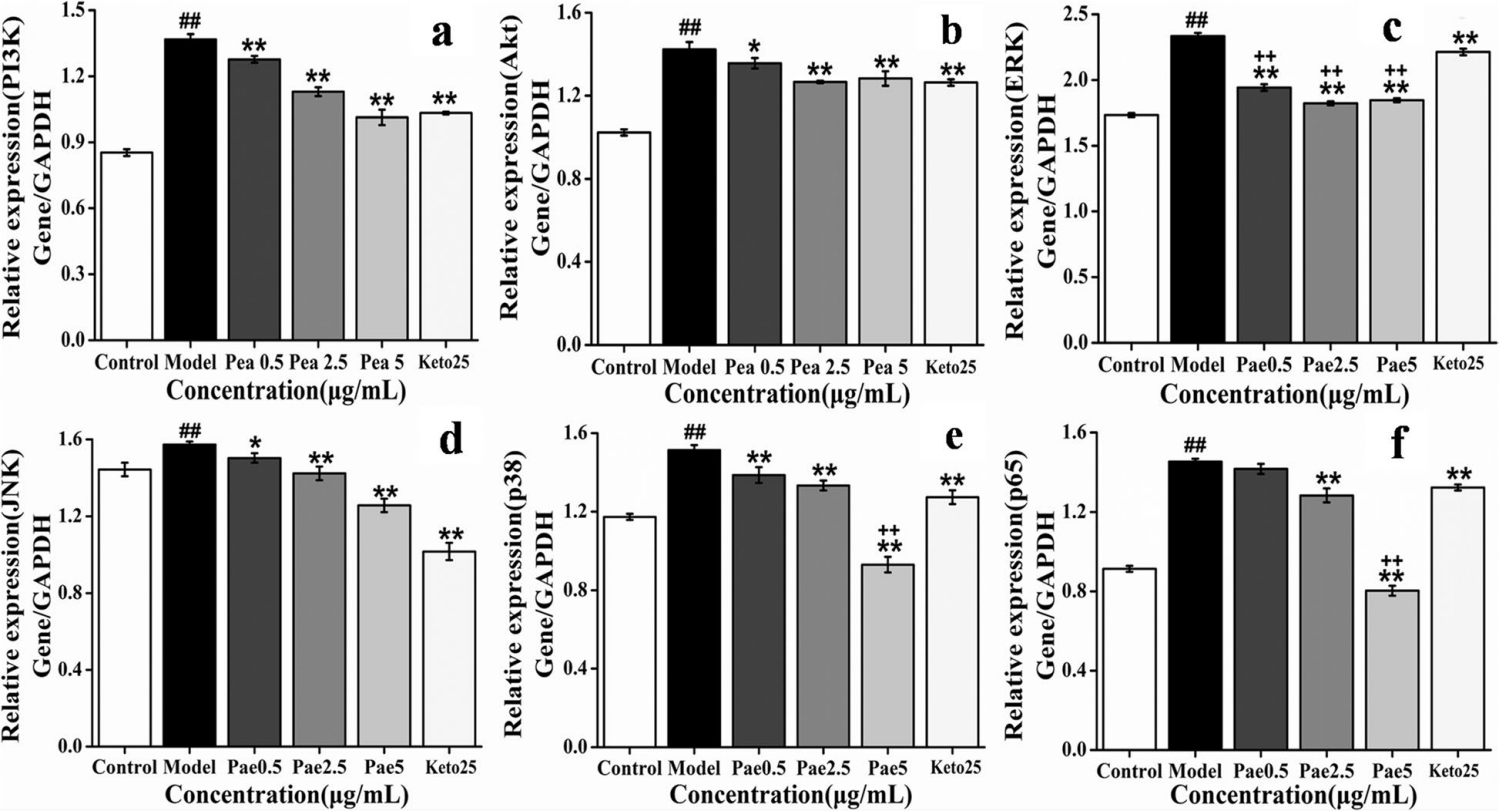
Effect of Pae on the expression of PI3K, Akt, ERK, JNK, p38 and p65 (*n* = 3). (**a**) PI3K; (**b**) Akt; (**c**) ERK; (d) JNK; (**e**) p38; (**f**) p65.^##^*p* < 0.01 vs control; *p < 0.05, **p < 0.01 vs model; ^++^*p* < 0.01 vs Keto

Pae can inhibit the expression of PI3K, Akt, ERK, JNK, p38 and p65 genes when the degranulation of RBL-2H3 cells in a dose-dependent manner (except Akt and ERK). The inhibitory effect of 5 μg/mL Pae on ERK, p38 and p65 was stronger than Keto group (Fig. 10).

## Discussion

The characteristics of multi-component, multi-target and the interaction of each component of TCM make it a complex system, and network pharmacology is a more comprehensive and systematic research technology that aims to reveal the complexity of biological systems, drugs and diseases, which has certain similarities with TCM, and is becoming a hot spot in TCM research [21]. Zhang Z Y [14] used the method of network pharmacology to obtain the key targets and possible mechanisms of Siwu Decoction to treat breast cancer, which provided a basis for the development of anti-breast cancer drugs. Changying J [15] successfully predicted the active ingredients and main targets of Qinghuo Rougan Decoction to treat uveit is through network pharmacology. Because network pharmacology is particularly suitable for reflecting and explaining the interaction of multi-component and multi-targets of TCM, it points out a novel direction for the modernization research of TCM, and is expected to bring novel opportunities for promoting the exploration of the multi-component mechanism of TCM and the development of modern TCM.

As one of the TCMs that can be used in dietary supplement, PLP has been found to have anti-inflammatory, anti-tumor and immune regulation effects. So it has been widely used to treat many diseases. PLP is often combined with other TCMs in the treatment of allergy. Shaoyao Gancao Decoction and Xiaoqinglong Decoction are classic prescriptions with anti-allergic effects and have good therapeutic effects, and both contain PLP. Therefore, it is speculated that PLP may have anti-allergic activity, but the mechanism of its treatment of allergy has not been fully understood. However, considering that PLP has the characteristics of multiple components and multiple targets based on the theory of TCM, experimental research alone cannot systematically reveal the biological mechanism of PLP anti-type I allergy, and the holistic characteristics of network pharmacology are suitable for this research. Different from previous studies, this research used network pharmacology to predict the efficacious ingredients and key mechanisms of PLP anti-type I allergy, and then conducted in vitro experiments for verification.

The TCMSP database contains 499 TCMs included in the Chinese Pharmacopoeia and their 29,384 components, 3311 targets and 837 related diseases. Each component provides pharmacokinetic data, as well as potential targets and related disease information, so that the relationship network of drug-target-disease can be obtained, which provides a new platform for the in-depth study of the pharmacological mechanism of TCM [22]. In order to obtain more accurate compounds for more in-depth research, we selected compounds with OB ≥ 30% and DL ≥ 0.18 as potential active ingredients, and obtained 29 main active ingredients and 157 targets of PLP, among which Pae is one of the main effective ingredients, which has high OB and DL values. Moreover, the existing research on PLP mainly focused on Pae, indicating that the data analysis has high reliability. GeneCards and OMIM databases are often used to screen disease-related targets. Using these two databases to search will help to obtain more comprehensive and detailed disease targets and improve accuracy. Through searching, we found 2424 targets related to ‘allergy’. GO and KEGG analysis are often used to analyze the function of target genes and related enrichment pathways. They are the most important data analysis in the network pharmacology system, and it is also a key step for network pharmacology to reveal the mechanism of drug to treat diseases [23]. By sorting out the intersection of targets, there are 50 possible targets for PLP anti-allergy. Through GO-BP analysis, the biological processes involved in the anti-allergic effect of PLP mainly include: positive regulation of cell biosynthesis, regulation of cell death and apoptosis, and intracellular signal cascades. GO-CC analysis showed that the cellular location of the anti-allergic effect of PLP mainly included the extracellular area and plasma membrane. GO-MF analysis showed that the molecular processes involved in the anti-allergic effect of PLP are antioxidant activity, MAPK activity, binding of Ca^2+^ and triglycerides and so on, among which the Ca^2+^ concentration is closely related to the occurrence of type I allergy. KEGG analysis obtained 31 related pathways of PLP anti-allergy, including the FcεR I signal pathway that is closely related to type I allergy, which researchers are familiar with, indicated that PLP has the potential to treat allergy, and also verified the reliability of network pharmacological analysis. The results concurrently showed that PLP may regulate allergy through signal pathways such as MAPK, TNF, PI3K/Akt, apoptosis and Th cell differentiation.

The obtained network of drug-target-pathway contains 52 nodes and 153 edges, among which MAPK 1, MAPK 10, MAPK 14 and TNF have high topological metrics and may be key targets. Combined with the results of KEGG analysis, it is found that these four important targets are distributed in the FcεR I signal pathway. MAPK 1, MAPK 10, and MAPK 14 belong to the MAPK family and are the integration points of many biochemical signals. They regulate cell proliferation, differentiation, and transcriptional regulation, and are closely related to multiple signal pathways involved in the regulation of allergy. TNF is related to various diseases such as allergy, autoimmune diseases, and tumors. Therefore, it is speculated that PLP may exert its inhibitory effect on allergy mainly through these targets and FcεR I signal pathway, and Pae, as the main component of PLP, may also inhibit the degranulation of mast cells (MC) by acting on these targets and pathways, and then play a therapeutic effect on type I allergy. Furthermore, the research on the chemical components and mechanism of PLP used for immune regulation and anti-inflammation is mainly focused on Pae [24, 25], so Pae was selected as the representative of PLP as the research object of subsequent in vitro experiments.

In addition to the OB values mentioned above. Studies have reported that the absorption permeability and absorption rate of Pae are approximately the same between various sites in the small intestine. And the absorption mechanism is passive diffusion. After oral administration of Pae, it is mainly absorbed in the form of metabolites of paeonimetabolin-I (PM-I) and paeoniflorgenin (PG). Shaoyao Gancao Decoction (a dose equivalent to Pae 25 mg/kg) was administered to rats, and the peak plasma concentrations (C_max_) of Pae and PM-I were 0.21 and 2.05 mg/L, respectively. In addition, the study also found that Baishao decoction (a dose equivalent to Pae 110 mg/kg) was administered to rats, and the C_max_ of PG was as high as 8 mg/L. The peak time (T_max_) of PM-I and PG were 3.0 h and 10 min, respectively. Pae has strong hydrophilicity, weak lipophilicity, and weak transmembrane absorption ability, but it can quickly reach the brain tissue through the blood–brain barrier. The mean AUC of Pae was 615.7 mg/min·L. Pae is less affected by liver metabolism, but can be degraded by glycosidases and anaerobic bacteria in intestine [26]. At present, drug research mostly focuses on the effect on the absorption of Pae, and there are few reports on the effect on the tissue distribution characteristics, metabolic pathways and metabolites of Pae.

RBL-2H3 cells possess the biological characteristics of MCs. And RBL-2H3 cells are used as the classic model for studying degranulation reaction in vitro. Therefore, after considering various factors, we finally chose RBL-2H3 cells as the cell model. To improve the reliability of the results, we chose Keto as the positive control drug. It has a strong anti-allergic effect, and can inhibit the release of allergic mediators from MCs and stabilize their membranes. Keto can also block Ca^2+^ channels and inhibit IgE synthesis. Thus, it is often used as a positive control drug in anti-allergy experiments.

According to different pathogenesis, allergy can be divided into 4 types, among which type I allergy is the most common in life [27]. The pathogenesis of type I allergy is complicated, and the specific and comprehensive regulation mechanism is still unclear. IgE/FcεR I is a classic signal pathway that directly regulates type I allergy. There are many studies on it, but the signal network that it participates in the development of type I allergy still needs to be perfected and supplemented. This study focused on the IgE/FcεR I signal pathway, and selected the other more important signal pathways in the results of network pharmacology for analysis, so as to prove the possible mechanism of PLP to treat type I allergy.

The classic IgE/FcεR I signal pathway includes Syk, Lyn and Fyn, among which Lyn and Syk as initial signals to participate in the activation of MC, and they have become key therapeutic targets for allergic diseases. Activated Syk can finally activate PLCγ and PI3K, which can cause the degranulation of MC [28, 29]. Fyn is the upstream of IgE/FcεR I signal pathway. The cross-linking of FcεR I can activate Fyn-dependent Gab2, and Gab2 can bind to PI3K, which will eventually activate Akt [30, 31]. In this study, the results of Western Blot and RT-qPCR showed that Pae can inhibit the phosphorylation of Lyn and Syk proteins and the expression of Lyn, Syk, Fyn, PLCγ, PI3K and Akt genes when the degranulation of MC. This result is consistent with the predicted results of network pharmacology, indicating that the network pharmacology method established in this study has good credibility, demonstrating that Pae can inhibit IgE/FcεR I and PI3K/Akt signal pathways.

When the IgE/FcεR I signal pathway is activated, it will directly or indirectly activate the MAPK and NF-κB signal pathways [32, 33]. MAPK includes JNK, ERK and p38 [34]. They mediate extracellular and nuclear signal transduction pathways, which can promote the activation of cytoplasmic phospholipase A2 and transfer to the cell membrane, thereby prompting MC to secrete biologically active mediators [35]. NF-κB is formed by p50 and p65, and is also closely related to MC degranulation [36]. Li L [37] found that allergy can be treated by inhibiting MAPK and NF-κB signal pathways. In this experiment, RT-qPCR was used to detect the effect of Pae on the expression of ERK, JNK, p38 and p65 genes when MC degranulation, showing that Pae can inhibit the expression of JNK, p38 and p65, but its inhibitory effect on ERK is weak, suggesting that Pae's inhibitory effect may be selective. These convincing evidences show that the mechanism of Pae on type I allergy is multi-target and multi-pathway, which is consistent with the experimental results of others we mentioned above. Our study revealed Pae has inhibitory effects on the key genes of in the downstream signal pathway of IgE/FcεR I, further confirming the multi-dimensional regulatory mechanism of Pae to treat allergy, which provides new support and reference for the study of the mechanism of PLP in the treatment of type I allergy.

## Conclusions

**Fig. 11:**
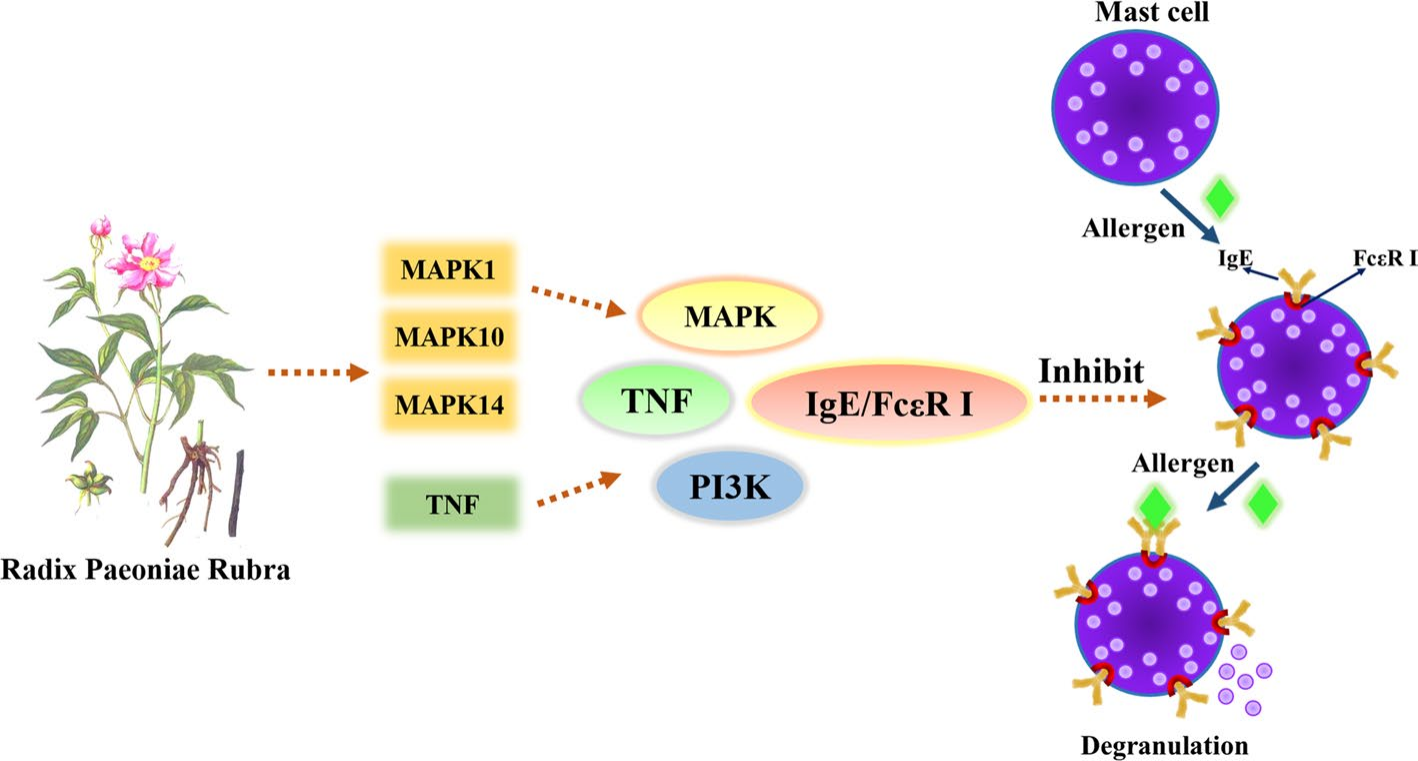
The provable mechanism of PLP anti-Type I allergy derived from this study

**Fig. 12:**
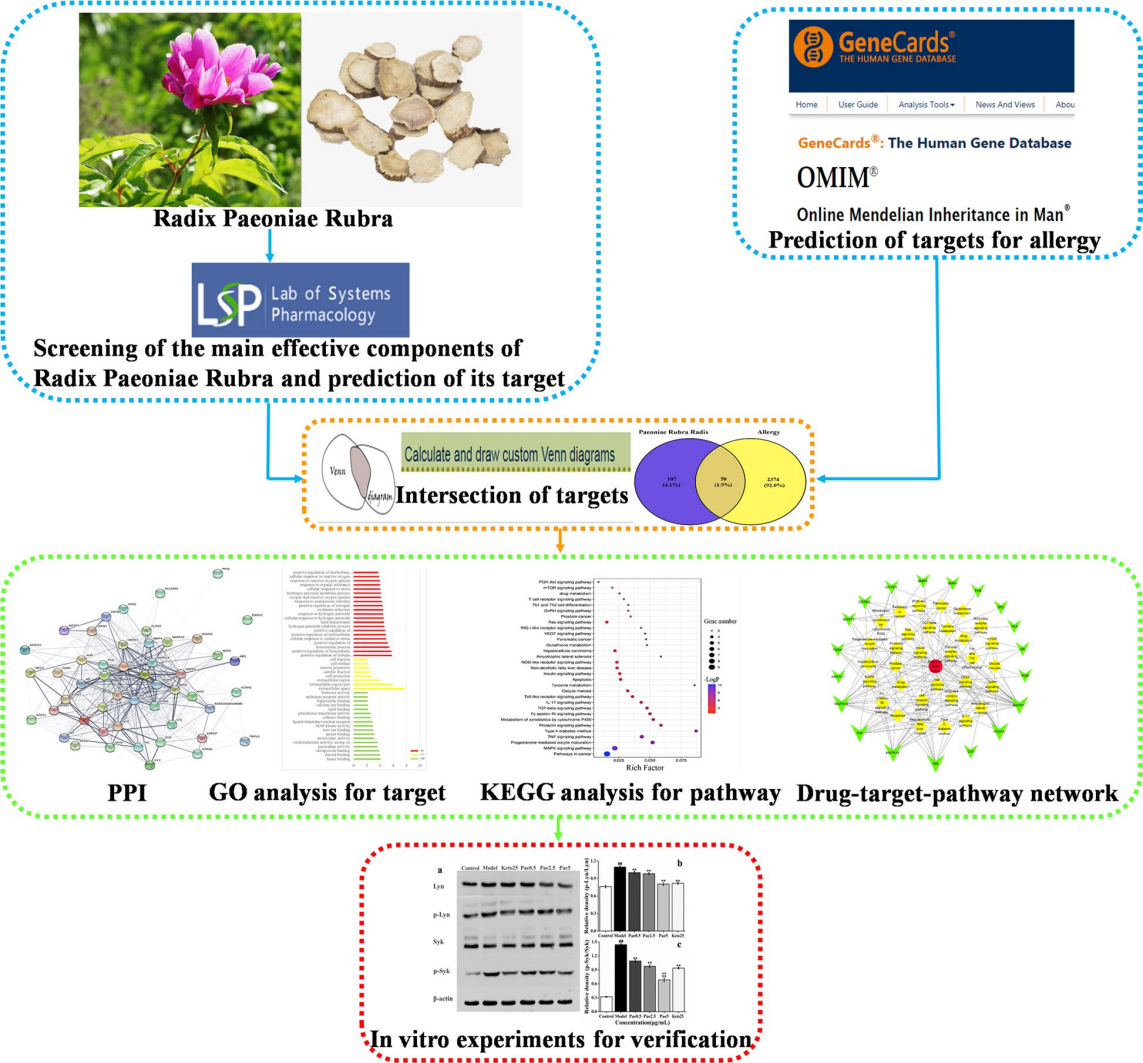
Graphical abstract of this paper

In summary, it was speculated that MAPK 1, MAPK 10, MAPK 14 and TNF may be the key targets of PLP to treat allergy. By interacting with these targets, PLP regulates FcεR I, MAPK, TNF, PI3K/Akt and Th cell differentiation and other signal pathways to participate in the occurrence and development of type I allergy (Fig. 11). Moreover, according to the results of Western Blot and RT-qPCR, Pae has been proven to have a therapeutic effect on type I allergy, which is achieved by regulating IgE/FcεR I and downstream signal pathways. These results of this study will offer a great opportunity for the deep understanding of the pharmacological mechanisms of PLP (Fig. 12). But there is no doubt that in order to fully reveal the mechanism of PLP and Pae, further in-depth research is needed. Further studies were planned where other cell and animal models related to type I allergy will be established to verify its inhibitory effect on type I allergy, which can provide a theoretical basis for the development of related fields and new drugs research.

## Supplementary Information

**Additional file 1: Fig.S1.** Original image of the expression of Lyn in RBL-2H3 cells detected by Western Blot.

**Additional file 2: Fig.S2.** Original image of the expression of p-Lyn in RBL-2H3 cells detected by Western Blot.

**Additional file 3: Fig.S3.** Original image of the expression of Syk in RBL-2H3 cells detected by Western Blot.

**Additional file 4: Fig.S4.** Original image of the expression of p-Syk in RBL-2H3 cells detected by Western Blot.

**Additional file 5: Fig.S5.** Original image of the expression of β-actin in RBL-2H3 cells detected by Western Blot.

## Data Availability

All data generated or analyzed during this study are included in this published article and its supplementary information files.

## Abbreviations

TCM: Traditional Chinese medicine
BP: Biological process
CC: Cell component
DL: Drug-likeness
GO: Gene Ontology
IgE: Immunoglobulin E
Keto: Ketotifen fumarate
KEGG: Kyoto Encyclopedia of Genes and Genomes
MC: Mast cells
MF: Molecular function
OB: Oral bioavailability
Pae: Paeoniflorin
PLCγ: Phospholipase C γ
PLP: Paeonia lactiflora Pall.
PPI: Protein–protein interaction
TCMSP: Traditional Chinese Medicine Systems Pharmacology Database and Analysis Platform
PM-I: Paeonimetabolin-I
PG: Paeoniflorgenin
