# Supplementary figures and images for: Network pharmacology-based analysis and experimental in vitro validation on the mechanism of Paeonia lactiflora Pall. in the treatment for type I allergy
Source: BMC Complement Med Ther. 2022 Jul 25;22:199. doi: 10.1186/s12906-022-03677-z (PMC9317138; doi:10.1186/s12906-022-03677-z)

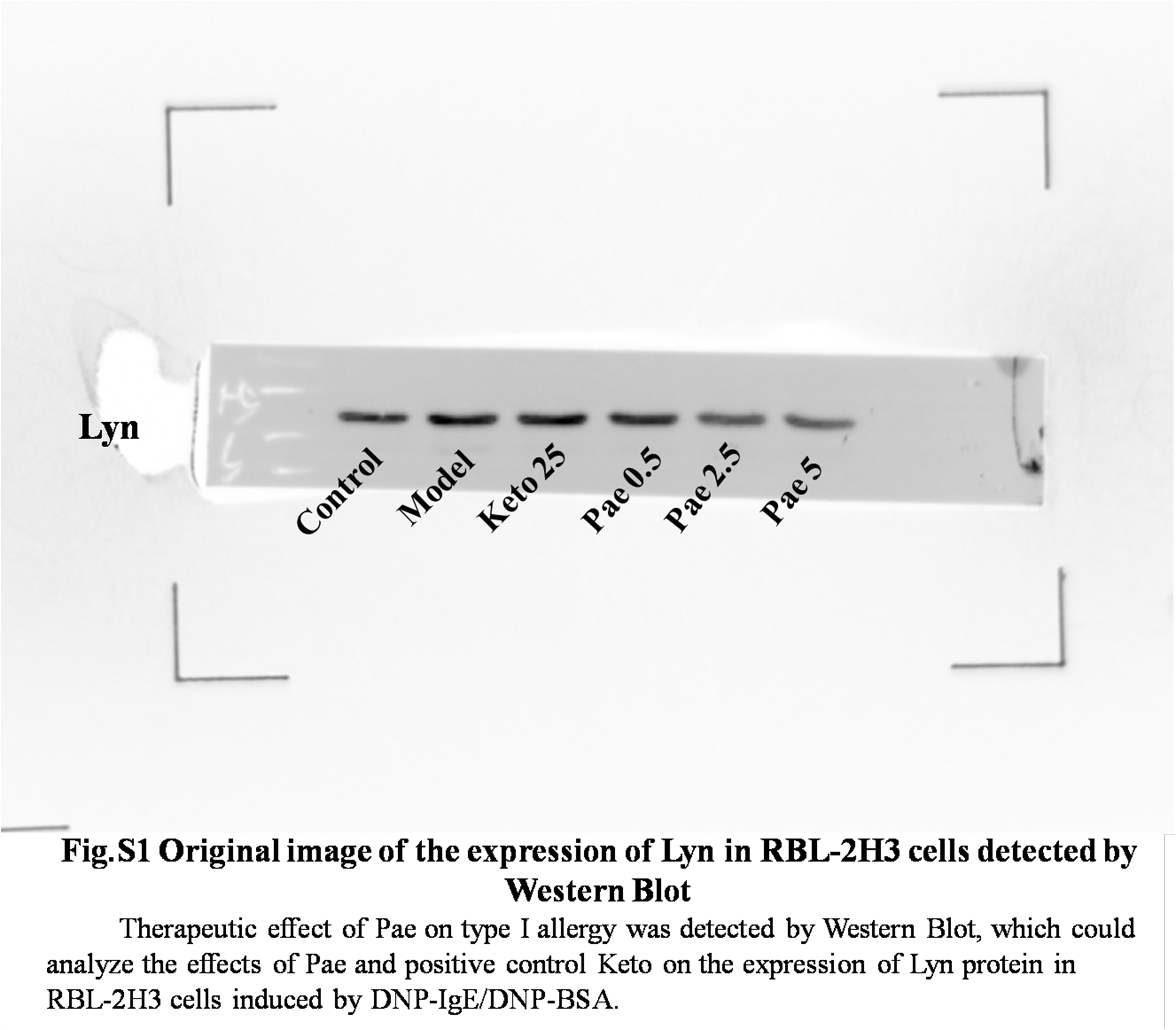

Supplement: Supplementary file 1 — Additional file 1: Fig.S1. Original image of the expression of Lyn in RBL-2H3 cells detected by Western Blot. [file 12906_2022_3677_MOESM1_ESM.tif]

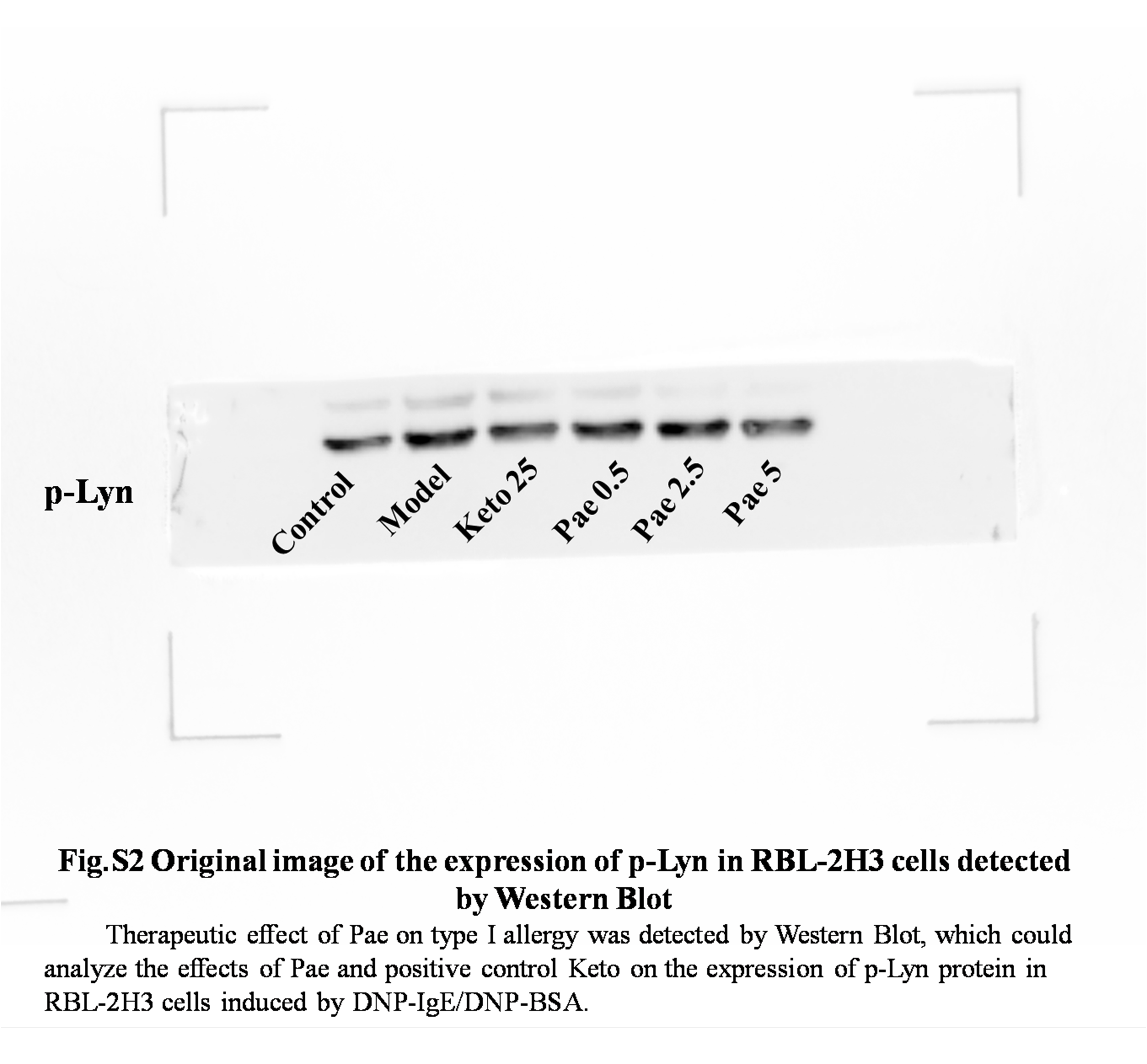

Supplement: Supplementary file 2 — Additional file 2: Fig.S2. Original image of the expression of p-Lyn in RBL-2H3 cells detected by Western Blot. [file 12906_2022_3677_MOESM2_ESM.tif]

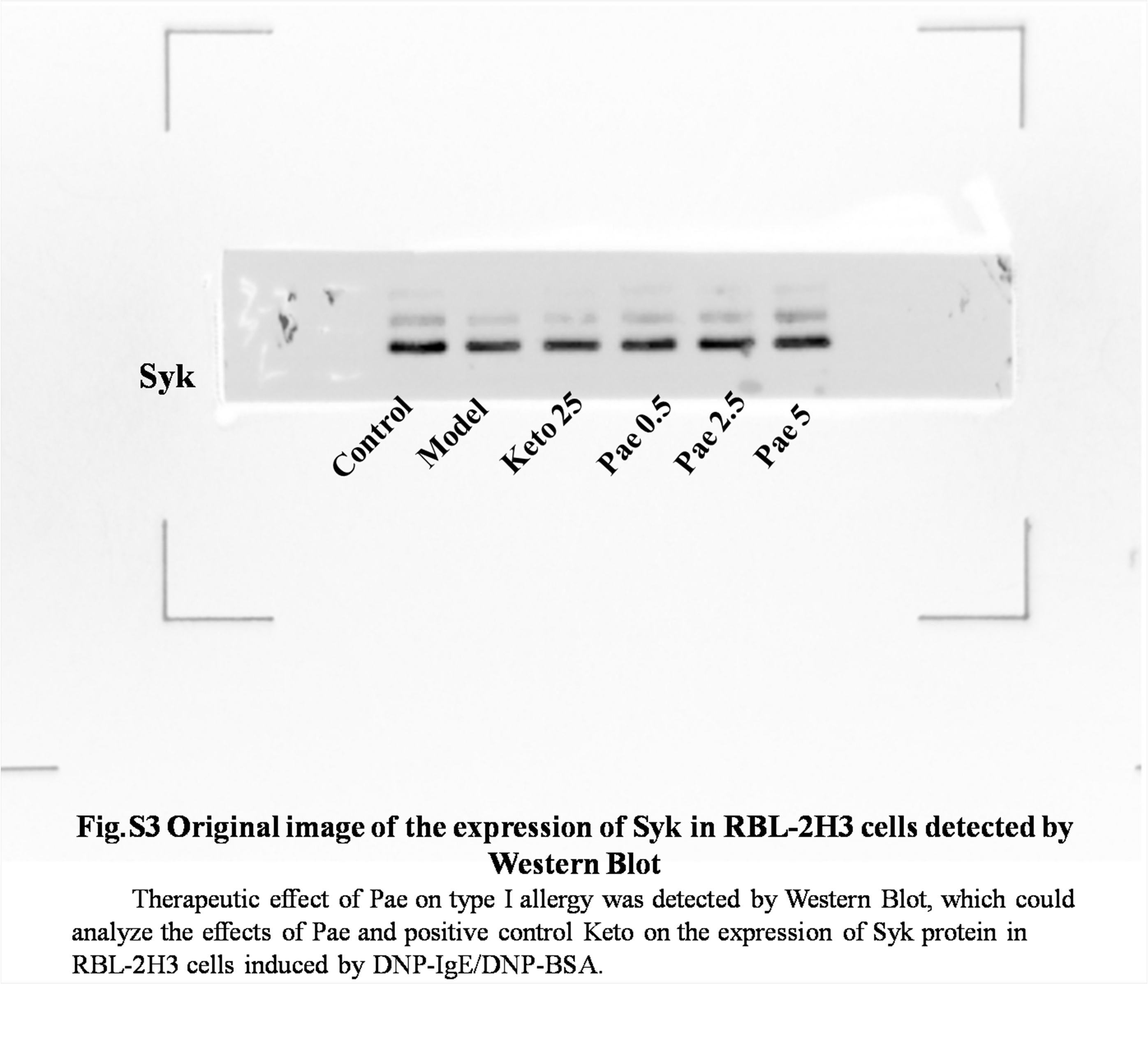

Supplement: Supplementary file 3 — Additional file 3: Fig.S3. Original image of the expression of Syk in RBL-2H3 cells detected by Western Blot. [file 12906_2022_3677_MOESM3_ESM.tif]

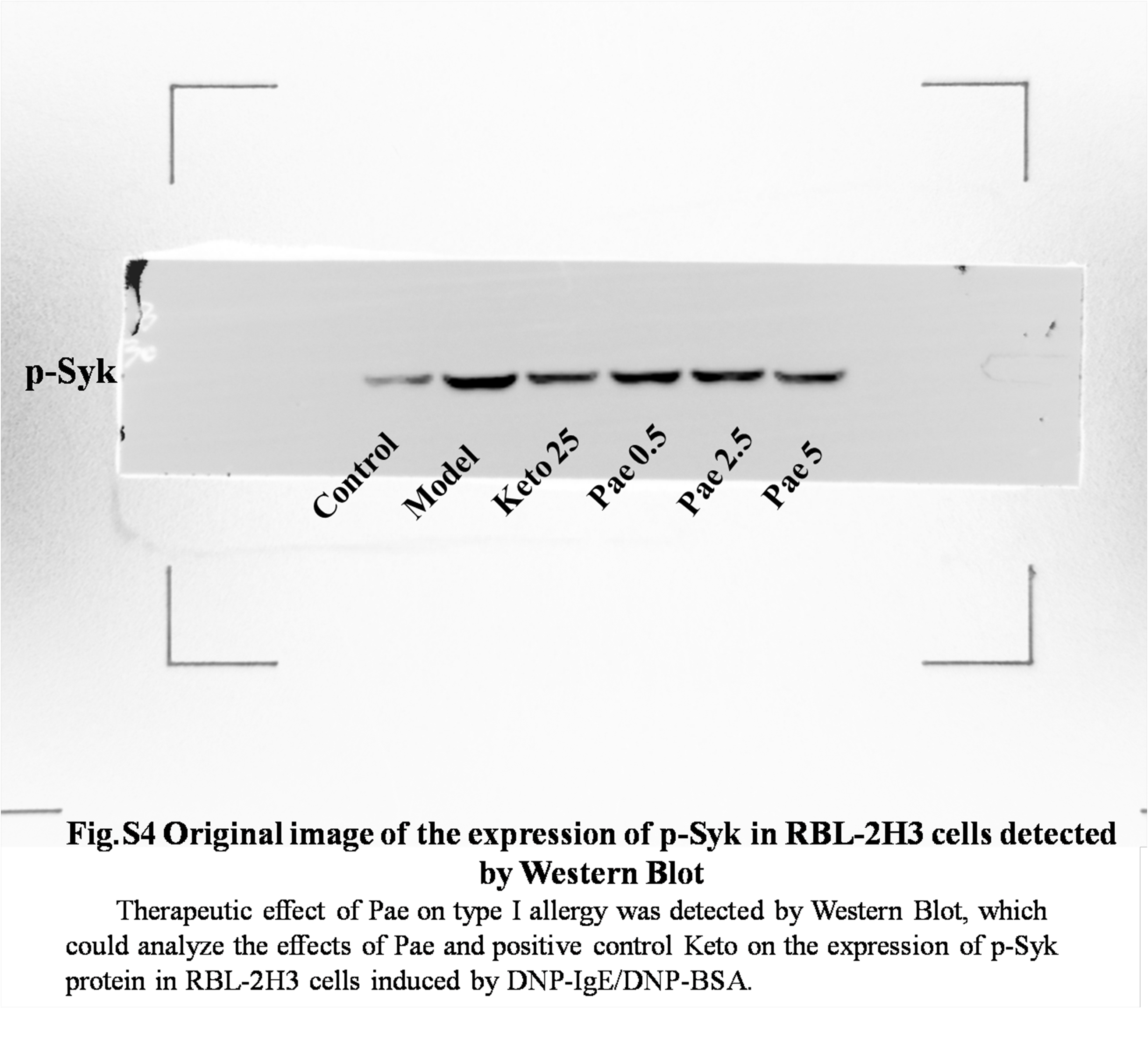

Supplement: Supplementary file 4 — Additional file 4: Fig.S4. Original image of the expression of p-Syk in RBL-2H3 cells detected by Western Blot. [file 12906_2022_3677_MOESM4_ESM.tif]

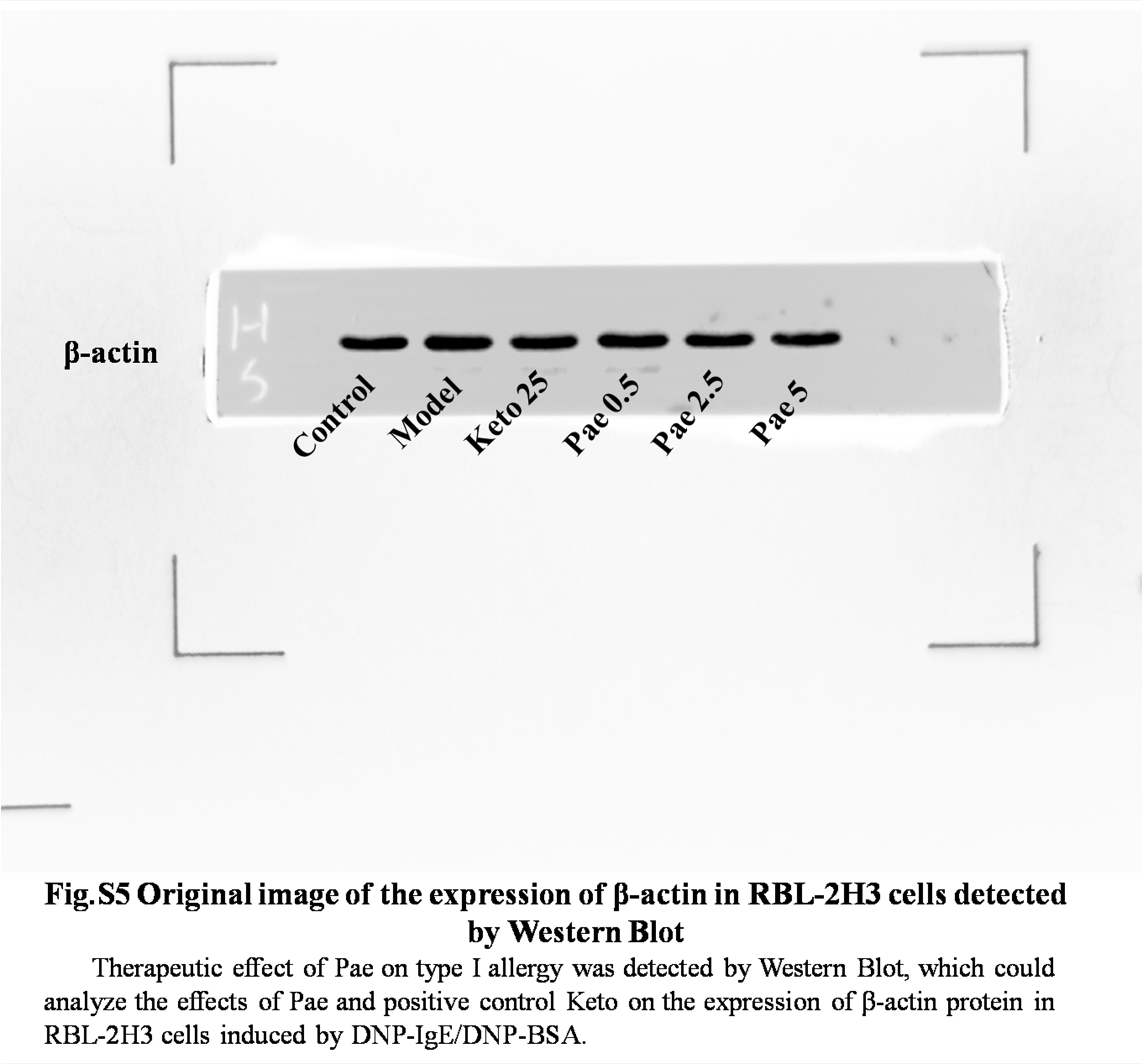

Supplement: Supplementary file 5 — Additional file 5: Fig.S5. Original image of the expression of β-actin in RBL-2H3 cells detected by Western Blot. [file 12906_2022_3677_MOESM5_ESM.tif]
